# Effect of Doping Temperatures and Nitrogen Precursors on the Physicochemical, Optical, and Electrical Conductivity Properties of Nitrogen-Doped Reduced Graphene Oxide

**DOI:** 10.3390/ma12203376

**Published:** 2019-10-16

**Authors:** Nonjabulo P. D. Ngidi, Moses A. Ollengo, Vincent O. Nyamori

**Affiliations:** School of Chemistry and Physics, University of KwaZulu-Natal, Westville Campus, Private Bag X54001, Durban 4000, South Africa; nonjabulongidi@gmail.com (N.P.D.N.); mosesollengo@gmail.com (M.A.O.)

**Keywords:** reduced graphene oxide, nitrogen-doping, chemical vapor deposition, physicochemical properties, optical properties, electrical conductivity

## Abstract

The greatest challenge in graphene-based material synthesis is achieving large surface area of high conductivity. Thus, tuning physico-electrochemical properties of these materials is of paramount importance. An even greater problem is to obtain a desired dopant configuration which allows control over device sensitivity and enhanced reproducibility. In this work, substitutional doping of graphene oxide (GO) with nitrogen atoms to induce lattice–structural modification of GO resulted in nitrogen-doped reduced graphene oxide (N-rGO). The effect of doping temperatures and various nitrogen precursors on the physicochemical, optical, and conductivity properties of N-rGO is hereby reported. This was achieved by thermal treating GO with different nitrogen precursors at various doping temperatures. The lowest doping temperature (600 °C) resulted in less thermally stable N-rGO, yet with higher porosity, while the highest doping temperature (800 °C) produced the opposite results. The choice of nitrogen precursors had a significant impact on the atomic percentage of nitrogen in N-rGO. Nitrogen-rich precursor, 4-nitro-*ο*-phenylenediamine, provided N-rGO with favorable physicochemical properties (larger surface area of 154.02 m^2^ g^−1^) with an enhanced electrical conductivity (0.133 S cm^−1^) property, making it more useful in energy storage devices. Thus, by adjusting the doping temperatures and nitrogen precursors, one can tailor various properties of N-rGO.

## 1. Introduction

Functionalization of carbon-based materials, such as graphene and carbon nanotubes for different purposes, is gaining a lot of attention in the field of material science. This interest arises because of their low cost, unique stable physicochemical properties, and broad applications. The former includes energy-harvesting devices [1], supercapacitors [2], sensors [3], field-effect transistors [4] and medical uses [5]. Certain extrinsic properties, such as electrical conductivity, high chemical stability, and a zero band-gap, enable some carbon-based materials to perform as semi-metals and semi-conductors [6]. Graphene, for instance, has a zero band-gap which needs to be manipulated for use in various applications such as solar cells. When graphene is chemically doped, it can change one absorbed photon and cause an increase in power conversion efficiency of solar cells [7]. However, graphene is a transparent material with a low coefficient of light absorption. Therefore, when graphene is applied in solar cells, it tends to produce a lower power conversion efficiency than solar cells based on heteroatom-doped graphene [7]. Thus, creating a well-tuned and sizeable band-gap to improve the coefficient of light absorption of graphene is a great challenge but with enormous interest.

The band-gap of graphene can be tuned by altering the surface chemistry through substitutional doping [8]. This can be achieved using selected heteroatoms to tune and enhance the band structure and conductivity [9,10]. Various heteroatoms that have commonly been employed in substitutional doping include boron [11,12,13] and nitrogen [14,15,16]. This is because they possess similar atomic radii and sizes to carbon and impact interesting electron chemistry within the graphene framework [17]. These heteroatoms have a significant effect on the electrical properties of graphene which is shown by a p-type conductivity for boron whereas nitrogen results in n-type conductivity [18]. In the case of strong p-type doping, it can be conferred by the interaction with the environment, hence, nitrogen-doping does not always confer n-type conductivity unless graphene is encapsulated [19]. The nitrogen atom is mostly used in chemical doping of graphene or graphene oxide (GO). This is because nitrogen atom acts as defect site in the crystal structure of graphene and these defective centers can enhance the electrochemical activity of graphene or GO [20].

Nitrogen-doping suppresses the density of state of graphene near the Fermi level and results to band-gap opening. Furthermore, nitrogen-doping tends to introduce strong electron donor states and leads to n-type or p-type semiconductor behavior depending on the bonding configuration. The conductivity and carrier mobilities of nitrogen-doped graphene are lower than pristine graphene due to the presence of nitrogen atom and defects introduced during the nitrogen-doping process which are capable of functioning as scattering centers that hinder the electron or hole transport [21]. Boron-doping in graphene, results in a p-type doping and is also highly favorable. This is because B-C bond is about 0.5% longer than the C-C bond while N-C bond is about the same as C-C bond in length, enabling formation of relaxed structure of boron-doped graphene. Boron-doping tends to introduce more holes into the valence band of graphene resulting in a high carrier concentration. Boron-doped graphene is reported to have high conductivity compared to pristine graphene [22] and nitrogen-doped graphene [23], due to the large density of state near the Fermi level.

Doping GO with nitrogen, results in nitrogen-doped reduced graphene oxide (N-rGO). The ideal physicochemical properties of N-rGO for optoelectrical applications include a large surface area and high chemical stability. These physicochemical properties of N-rGO can be significantly enhanced by improving the atomic percentage of nitrogen [24] and the bonding configuration [25]. Various bonding configurations of nitrogen in N-rGO have been reported, e.g., pyrrolic-N [25], pyridinic-N [26], quaternary-N [27,28] and oxide-N [29]. These bonding configurations impart various effects on the carrier concentration which tend to produce well-defined band structures in doped GO [30].

The mechanism of formation of N-rGO is still a fascinating phenomenon because it is not well understood and there is more that can be done to manipulate it. Therefore, synthetic procedures for N-rGO need a certain level of control regarding the required extent of doping and the bonding configuration of nitrogen. Different synthetic approaches have been employed in the in-situ synthesis of N-rGO, such as arc discharge [31], plasma method [32], thermal annealing [33] and chemical vapor deposition (CVD) [34,35]. The CVD approach is mostly preferred because it is easier to scale-up and produces relatively high-quality N-rGO. Scientific reports on the synthesis of N-rGO via the CVD approach indicate that the mostly used materials are metal catalyst (Cu, Ni, Co or Fe) [36,37] and organic molecules [38].

In the CVD synthesis of N-rGO, several factors including the type of carrier gas, doping temperature and nitrogen precursor (used either as a solid, liquid or in the gaseous phase), influence the nitrogen content and properties of the final product [39,40]. Nang et al. [41] and Panchakarla et al. [42] reported the use of dimethylformamide and pyridine, respectively, as liquid nitrogen precursors for the synthesis of N-rGO, with the former achieving a very low nitrogen content of 0.64%. The drawback of liquid nitrogen precursors is that they are expensive, dangerous, and highly flammable when used in the CVD method.

The alternative to liquid and gaseous nitrogen precursors is solid nitrogen precursors. The use of solid nitrogen precursors, such as monoethanolamine [43], urea [33,44], 1,3,5-triazine [24], pentachloropyridine [36] and the combination of imidazole and melamine [45] have been reported and observed to result in high doping levels. Lu et al. [24] reported the CVD synthesis of a few-layered nitrogen-doped graphene oxide containing atomic percentages of between 2.1 and 5.6% nitrogen by making use of the carbon and nitrogen precursor 1,3,5-triazine and Cu foil as catalyst. Doped graphene films with a higher nitrogen content of approximately 5.6% were obtained at a doping temperature of 990 °C, with melamine [46] as a solid nitrogen precursor. The use of the solid nitrogen precursor, pentachloropyridine, in the synthesis of nitrogen-doped graphene was reported by Wan et al. [36] to yield a nitrogen content between 4.4 and 7.5%. Solid nitrogen precursors are cost-effective and are easy to handle compared with liquid and gaseous nitrogen precursors.

**Figure 1:**
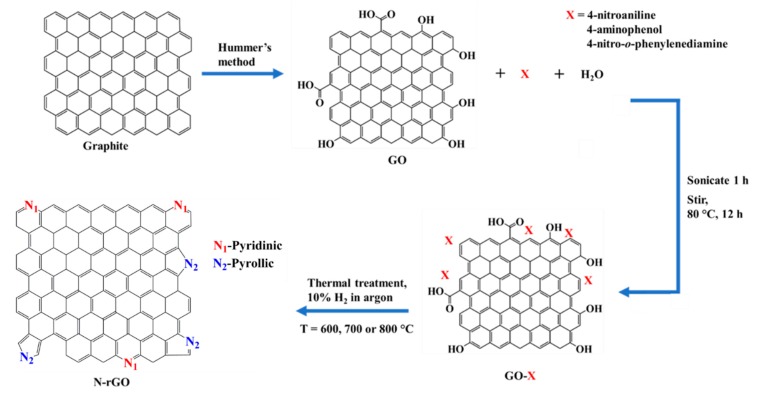
Schematic diagram of the conversion of graphite to N-rGO.

In this work, we report for the first time, the effect of different doping temperatures and solid nitrogen precursors on the physicochemical (nitrogen content, crystallinity, thermal stability and bonding configuration), optical (band-gap energy and charge recombination) and electrical conductivity properties of N-rGO. The synthesis of N-rGO (Figure 1) was achieved by liquid exfoliation of GO, high temperature vapor reduction of GO and doping it with nitrogen atoms from various solid nitrogen precursors (4-nitroaniline, 4-aminophenol and 4-nitro-*ο*-phenylenediamine). These nitrogen precursors were chosen because they possess different number of nitrogen atoms on their structures or frameworks, and therefore the effect of the number of nitrogen atoms contained in the nitrogen precursor was also investigated. 

## 2. Materials and Methods

### 2.1. Materials and Instrumentation 

Graphite powder (99.99% -trace metal basis), sodium nitrate (99%), potassium permanganate (99%), 4-nitroaniline (≥99%), 4-aminophenol (≥99%) and 4-nitro-*ο*-phenylenediamine (≥99%) were purchased from Sigma-Aldrich, Johannesburg, South Africa. These chemicals were of analytical grade and were used without further purification. Hydrogen peroxide (100%) was purchased from Merck Laboratory Supplies, Johannesburg, South Africa. Sulfuric acid (98%) was purchased from Promark Chemicals, Johannesburg, South Africa. Double distilled water was obtained from a double distiller, Glass Chem water distiller model Ws4lcd was supplied by Shalom Laboratory Supplies, Durban, South Africa. A gas mixture of 10% hydrogen in argon (v/v) was purchased from Afrox Limited Gas Co., Durban, South Africa. Weighing of N-rGO was done on an electronic weighing balance, Mettler AE 200, Mundelein, IL USA. Ultrasonication was performed in a digital ultrasonic water bath (400 W) obtained from Shalom Laboratory Supplies, Durban, South Africa. 

### 2.2. Synthesis of N-rGO

A modified Hummer’s method was used to synthesize GO [47]. In brief, approximately 1 g of graphite powder and 1 g of sodium nitrate were mixed with 50 mL of concentrated sulfuric acid in a 500 mL round-bottom flask placed in an ice-bath and stirred for 30 min. After that, 6 g of potassium permanganate was added slowly to the mixture with the temperature kept at 5 °C to prevent explosion and excessive heating. Thereafter, the mixture was stirred for 3 h at a temperature of 35 °C and then further treated with 200 mL of 3% hydrogen peroxide while stirring for 30 min. The resulting GO was washed with double distilled water until a pH of 6 was achieved. The product was then filtered and dried in the oven for 24 h at a temperature of 80 °C.

The synthesized GO was simultaneously reduced (using 10% hydrogen in argon as a reducing agent) and doped with different nitrogen precursors, namely 4-nitroaniline, 4-aminophenol and 4-nitro-*ο*-phenylenediamine. This was done by mixing 70 mg of GO and 30 mg of nitrogen precursor in 50 mL double distilled water, followed by sonication (25 °C) for 1 h. The mixture was further stirred and heated for 6 h at a temperature of 100 °C to remove the excess water. After drying, the resulting black solid was heat-treated in a ceramic quartz boat placed in a tube furnace (Elite Thermal Systems Ltd., Model TSH12/50/160) in a mixture of 10% hydrogen in argon (v/v) at a flow rate of 100 mL min^−1^. The doping temperature of the furnace was set at each of 600, 700 and 800 °C, for each nitrogen precursor. The carrier gas flow rate and the doping temperature were maintained constant throughout the synthesis period of 2 h. After 2 h, the furnace was allowed to cool naturally to room temperature and N-rGO was collected and subsequently characterized.

### 2.3. Physicochemical Characterization 

The surface morphology of N-rGO was investigated by field emission scanning electron microscopy (FE-SEM, Carl Zeiss Ultra Plus, Cambridge, UK). Briefly, the aluminum stub sample holders were coated with piece of a sticky carbon tape; after that the N-rGO was sprinkled on the carbon tape and gold coated thrice before SEM analysis. The microstructural features of the N-rGO were evaluated by means of high-resolution–transmission electron microscopy (HR-TEM, JOEL JEM model 1010, Peabody, MA, USA), and set at an accelerating voltage of 100 kV at different magnifications. 

The crystallinity or graphitic nature of the N-rGO was investigated with a Delta Nu Advantage 532^TM^ Raman spectrometer (Laramie, WY, USA) equipped with NuSpec^TM^ software (1.0., Microsoft Publisher, Redmond, WA, USA) and operated at a wavelength (λ) of 514.5 nm. The functional groups present in the N-rGO were investigated with a PerkinElmer Spectrum 100 Fourier transform infrared (FTIR) spectrometer (Akron, OH, USA) equipped with an attenuated total reflectance (ATR) accessory. Approximately 0.22 g of the N-rGO was pressed into a pellet for about 2 min, under a pressure of 10 Tons. The pellets were then placed on the diamond crystal for analysis. 

The thermal stability of N-rGO was measured with a TA Instruments Q series^TM^ thermal analysis instrument (DSC/thermogravimetric analysis (TGA) (SDT-Q600), New Castle, PA, USA) in air flowing at a rate of 50 mL min^−1^ and heated from room temperature up to 1000 °C at a ramping rate of 10 °C min^−1^. N-rGO were further characterized by X-ray photoelectron spectroscopy (XPS, Quantum 2000 with an X-ray source of monochromatic Al K_α_ (1486.7 eV), Chanhassen, MN, USA) to investigate the surface chemical composition of carbon and nitrogen.

A Micromeritics Tristar II 3020 surface area and porosity analyzer (Norcross, GA, USA) was used to determine the textural properties of N-rGO. Typically, a mass of approximately 0.1 g of N-rGO was degassed at 90 °C for 1 h, the temperature was then raised to 160 °C and the sample further degassed for 12 h using Micromeritics Vacprep 061 (sample degas system), before fitting N-rGO in the Micromeritics Tristar II instrument for analysis. The textural properties of the N-rGO were investigated at a temperature of −196 °C with N_2_ as the adsorbate. The specific surface areas were calculated with the Brunauer, Emmett and Teller (BET) model and the pore volumes were obtained by applying the Barrett-Joyner-Halenda (BJH) model.

The phase characteristics of the synthesized N-rGO were determined by X-ray powder diffraction (XRD, Rigaku/Dmax RB, The Woodlands, TX, USA) and the measurements were performed with graphite monochromated high-density with a 𝜃-𝜃 scan in locked coupled mode, using a Cu k_α_ radiation source (λ = 0.15406 nm). The absorbance of N-rGO was recorded with an ultraviolet-visible spectrophotometer (UV-Vis, Shimadzu, UV-1800, Roodepoort, South Africa). The GO and N-rGO were first dispersed in absolute ethanol and then sonicated for 30 min before UV-Visible spectrophotometric analysis. Charge recombination analysis of N-rGO was investigated with a PerkinElmer LS 55 spectrofluorometer (Akron, OH, USA) fitted with solid sample accessory. Excitation was performed at 310 nm, and the emission spectrum recorded from 450 to 550 nm with an excitation slit and emission slit at 5 nm and 2 nm, respectively (slid position). Electrical conductivity of N-rGO was determined by four-point probe (Keithley 2400 source-meter, Beaverton, OR, USA) measurements which were carried out on pellets with a thickness of 0.2 mm formed from N-rGO (0.03 g). 

## 3. Results and Discussion 

The physicochemical characteristics of N-rGO synthesized with different nitrogen precursors and at different doping temperatures of 600, 700 and 800 °C are presented. Both factors influence the level of nitrogen-doping, morphology, crystallinity, thermal stability, optical, and electrical conductivity properties of N-rGO.

### 3.1. Morphology 

**Figure 2:**
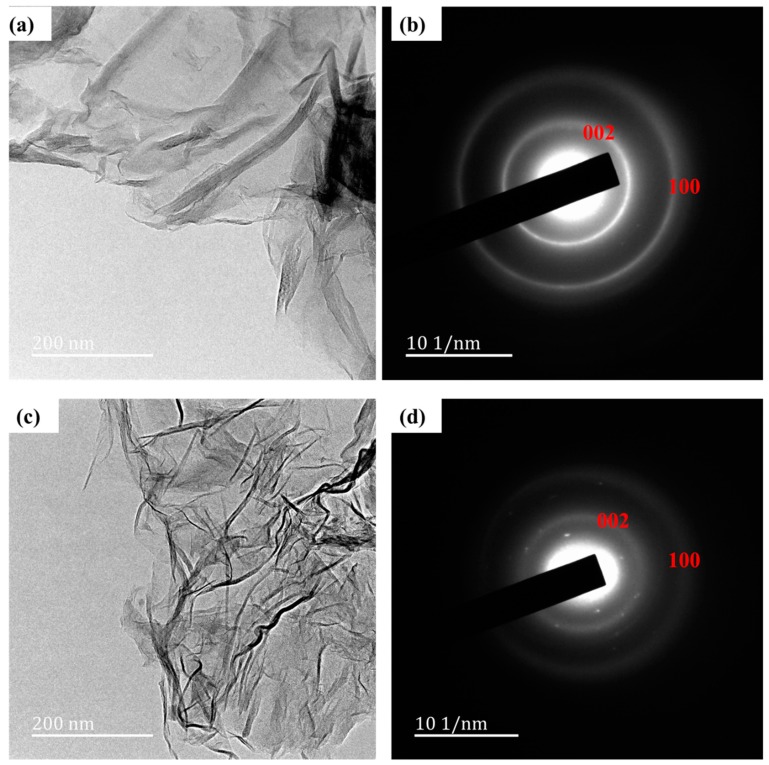
HR-TEM image of (**a**) GO, (**b**) SAED pattern of GO, (**c**) HR-TEM image of N-rGO-1N-600 °C and (**d**) SAED pattern of N-rGO-1N-600 °C.

The nitrogen atoms from the different nitrogen precursors were successfully introduced into the GO lattice. N-rGO synthesized from: 4-aminophenol is represented by N-rGO-1N, 4-nitroaniline is represented by N-rGO-2N while 4-nitro-*ο*-phenylenediamine is represented by N-rGO-3N. N-rGO in the SEM images showed a thick and overlapping sheet structure (Supplementary Materials—Figure S1). This was attributed to flake-like structures. A similar observation for SEM images of N-rGO was reported by Jiang et al. [48]. Further details of the structures were evaluated by HR-TEM. For comparison, Figure 2 presents the HR-TEM images and the selected area electron diffraction (SAED) patterns of GO and N-rGO. Both GO and N-rGO exhibited a wrinkled structure (Figure 2a), which increased after doping (Figure 2c). The more wrinkled structure in N-rGO is caused by the stimulation of defects such as pores, holes and cavities which were introduced during the process of doping [49]. The different doping temperatures and nitrogen precursors revealed no effect on the morphology of N-rGO.

The SAED patterns recorded to study the crystalline structures of GO and N-rGO revealed two diffraction rings which were associated with the (002) and (100) planes for both GO (Figure 2b) and N-rGO (Figure 2d). The presence of hexagonal diffraction spots in the electron diffraction pattern observed, indicates that N-rGO has a well-ordered structure while the occurrence of structural distortion after doping as revealed by the ring-like diffraction pattern. The observed disorder might be due to the introduction of functional groups, and the overlapping graphene sheets [50]. The diffraction spots in hexagonal positions in N-rGO are reflective of the preservation of the original honey-comb-like atomic structure of graphene [51]. The GO also shows a ring-like structure (distortion) which is due to ring spacing (Figure 2b).

**Figure 3:**
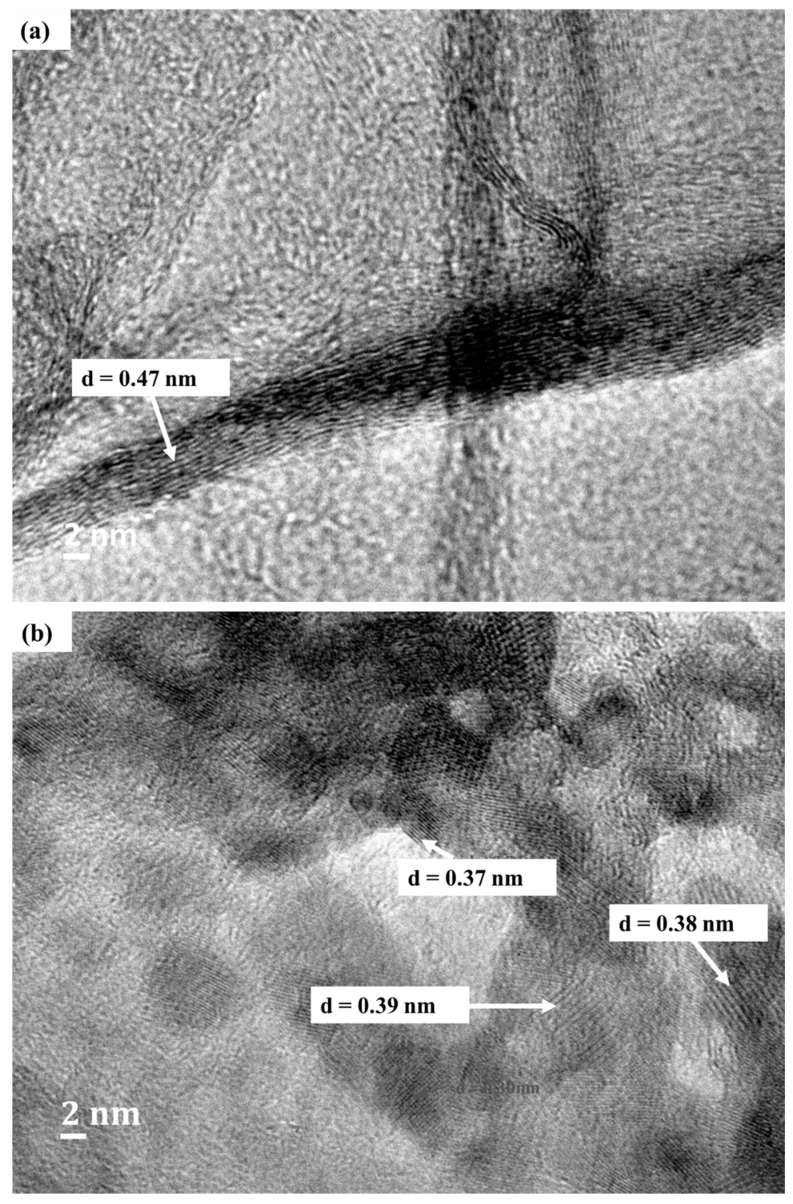
The d_002_ interlayer spacing of (**a**) GO and (**b**) N-rGO-1N-600 °C.

The different interlayer spacings (d_002_ spacing) on the edge of GO and at the cross-sections of layers of N-rGO as observed in the HR-TEM images (Figure 3a,b) showed that the carbon atom layers were not identical; this is indicative that the N-rGO consists of few-layers of graphene sheets (Table 1). After nitrogen-doping, the d_002_ spacing was found to decrease. For example, the d_002_ spacing of N-rGO-1N-600 °C was found to be 0.37 nm which is smaller than that of GO (0.47 nm). The decrease in d_002_ spacing after nitrogen-doping was attributed to the reduction of oxygen functional groups such as carboxyl, epoxy and hydroxyl groups [52]. 

The d_002_ spacing of N-rGO varied with different doping temperatures and nitrogen precursors used. The d_002_ spacing was found to increase with an increase in doping temperature. For instance, N-rGO-1N-600 °C, N-rGO-1N-700 °C and N-rGO-1N-800 °C have d_002_ spacings of 0.39, 0.40 and 0.44 nm, respectively. The smaller d_002_ spacing observed at a lower doping temperature (600 °C) is attributed to structural strains and the less crystalline nature of N-rGO. A larger d_002_ spacing was observed for N-rGO-1N synthesized from the nitrogen precursor, 4-aminophenol, at 800 °C. The interlayer spacing increased due to the distortion introduced by the inclusion of nitrogen. Such intercalation structural distortion has been widely reported for different carbon nanomaterials, such as carbon nanotubes [53]. Therefore, Raman spectroscopy was further used to investigate the effect of various temperatures and nitrogen precursors on the graphitic nature (structural properties) of N-rGO.

### 3.2. Structural Properties

The structural and electronic properties of N-rGO were investigated by Raman spectroscopy. Two major peaks were observed, namely the G-band peak (between 1580 and 1606 cm^−1^) which originates from the Raman 𝐸_2𝑔_ mode, and the D-band peak (between 1347 and 1363 cm^−1^) which is the disorder-induced band. The intensities of the D-band of N-rGO-3N-600 °C and N-rGO-3N-700 °C; and the G-bands of N-rGO-1N-600 °C and N-rGO-1N-700 °C, are of the same value (Table 2). However, their I_D_/I_G_ ratios are different. This indicates that the nitrogen dopant distribution in N-rGO was not homogeneous [54]. 

The G-bands for all N-rGO samples, showed a slight shift in frequency for all doping temperatures. A shift in the D-band (from 1354 to 1363 cm^−1^) of N-rGO-1N was observed as the doping temperature increased. This was due to the change in the bond length and symmetry of the C–C and C=C bonds in the graphene lattice and compressive stress on graphene during the annealing process [55]. The asymmetric line shape and shift of the G-band (from 1601 to 1606 cm^−1^) of GO and N-rGO-3N-600 °C, may be due to the increase in the percentage of nitrogen incorporated. Results showed that the changes in the D- and G-bands are associated with the increase in defects/dopant concentration.

It has been shown that relaxation or the change of lattice constant is highly asymmetric with lattice constant increasing by 0.32% with 2% in boron substitution and decreases very slightly with N substitution [42]. Panchakarla et al. [42] have shown that the inter-planar separation reduces by almost 2.7% in B-doped bilayer reduced graphene oxide while it remains almost unchanged in N-doped bilayers. However, there is a resultant large decrease and a slight increase in frequency shift in G-band with either B or N substitution. Boron affords a homogeneous distribution as such, disorder or the number of possible configurations increases with the concentration of dopant atoms and result in more prominent peaks of the D-band compared to nitrogen. However, G-band stiffens both with boron and nitrogen-doping and the intensity of the D-band is higher with respect to that of the G-band in all the doped samples. 

The intensities of the G- and D-bands differ, and this is evident in the I_D_/I_G_ ratio of N-rGO (Table 2). The I_D_/I_G_ ratio is an indication of the degree of disorder and graphitic nature of N-rGO. A broader width of the D-band, narrower width of the G-band and a larger I_D_/I_G_ ratio, suggest that N-rGO possesses many defective sites and different bonding structures (e.g., C-O, C-N) in the graphene lattice [56]. The I_D_/I_G_ ratios of all N-rGO decreased with increase in doping temperature (from 600 to 800 °C) for the same nitrogen precursor. The larger I_D_/I_G_ ratios observed at the lowest doping temperature of 600 °C imply a higher level of disorder (lower crystallinity). The highest doping temperature of 800 °C resulted in highly crystalline N-rGO because more amorphous products in N-rGO were reduced. Similar observations were reported by Capasso et al. [57]. Table 2 also shows the increase in defects upon an increase in nitrogen content (nitrogen precursor) in the graphene oxide lattice. In the case of N-rGO-3N that was obtained from a nitrogen precursor with the largest number of nitrogen atoms, a marked shift in the G-band was observed. This due to the increase in nitrogen atoms introduced in bond formation within the *sp*^2^ carbon lattice of the GO. N-rGO-3N samples were observed to be less crystalline, with I_D_/I_G_ ratios of 1.77, 1.40 and 0.88 at doping temperatures of 600, 700 and 800 °C, respectively. While other N-rGO-1N and N-rGO-2N samples which were synthesized from 4-aminophenol and 4-nitroaniline, respectively, were more crystalline. 

Apart from the characteristic D-band and G-band, GO and N-rGO have a third peak; 2D-band. The 2D band represents the second order of the D-band, which is alluded to as an overtone of the D-band. Its occurrence is due to two phonon lattice vibrational processes; however, it is not associated with defects, like D-band. Therefore, the 2D-band is regarded as a strong band in graphene even when there is no presence of the D-band. The observed 2D-band peak of GO had higher intensity compared to N-rGO, 2D-band. The intensity ratios of the G-band and 2D-band (I_2D_/I_G_ ratio) have been used to investigate the electron concentration of the N-rGO. Results showed that the I_2D_/I_G_ ratio changes as the number of nitrogen atoms in the graphene lattice increases (Table 2). The different nitrogen precursors have different nitrogen atoms insertion capacity into rGO thus, the change in I_2D_/I_G_.

The I_D_/I_G_ ratio of all N-rGO tends to increase as the I_2D_/I_G_ ratio decreases. A similar trend was also reported by Zafar et al. [54]. This is because N-rGO consists of extra scattering effect that arises from nitrogen induced electron doping. The 2D band is mostly dependent on the electron/hole scattering rate which is influenced by lattice and charge carrier doping. Therefore, the I_D_/I_G_ ratio would increase the electron-defect elastic scattering rate, while the I_2D_/I_G_ ratio would increase the electron-electron inelastic scattering (Coulomb interaction). However, the evaluation of doping level of N-rGO using the I_2D_/I_G_ ratio and blue-shifting of G-band is complicated because the G-band and 2D-band features are greatly affected by strains, defects, and number of layers. 

The crystallite size (La) of N-rGO, which depends on the I_D_/I_G_ ratio, was calculated with the aid of an equation reported by Mallet-Ladeira et al. [58] (which is an alternative to the Tuinstra–Koenig (TK) law given in Equation (1):

(1) HWHM=71−5.2 La

where *HWHM* stands for the half width at half maximum which is the half of the full width at half maximum (FWHM) when the function is symmetric. The crystallite size decreases remarkably with an increase in the I_D_/I_G_ ratio of N-rGO. N-rGO synthesized at the lowest temperature (600 °C) produced N-rGO with smaller crystallite size than that prepared at the highest temperature (800 °C). The crystallite sizes of N-rGO vary with the type of nitrogen precursor used. A smaller crystallite size of (2.49 nm) was observed in N-rGO-3N while N-rGO-2N and N-rGO-1N had a crystallite size of 4.07 and 4.20 nm, respectively at 600 °C. The crystallite size of GO (5.37 nm) was larger than that of N-rGO which indicates that thermal treatment and the type of nitrogen precursor affected the crystallite size of N-rGO. Previous studies have reported a link between the increase in I_D_/I_G_ ratio and smaller crystallite size which is due to the formation of small crystals during reduction [50]. 

### 3.3. Thermal Stability

**Figure 4:**
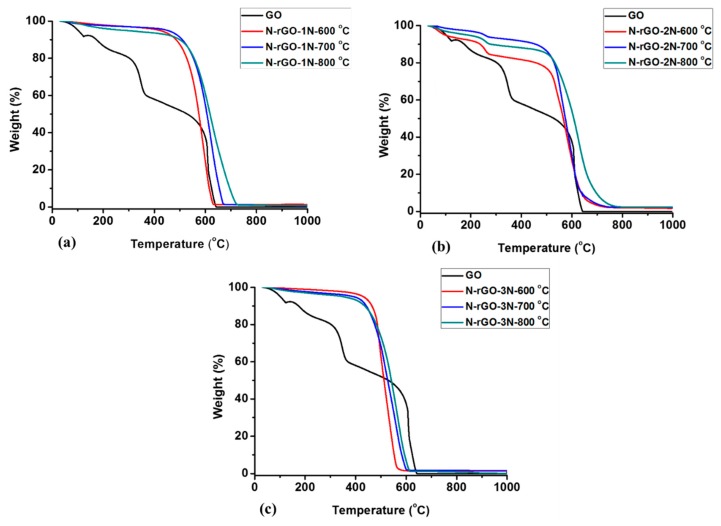
TGA thermograms of (**a**) N-rGO-1N, (**b**) N-rGO-2N and (**c**) N-rGO-3N.

The decomposition behavior of N-rGO was investigated by the thermogravimetric analysis (TGA). The synthesized N-rGO exhibited different thermal stabilities. TGA weight loss curves of N-rGO-1N, N-rGO-2N, and N-rGO-3N are shown in Figure 4a, Figure 4b, and Figure 4c, respectively.

The thermogram of GO showed a sequence of reaction steps because of the different oxygen-containing functional groups present in GO. These include carbonyl (C=O), hydroxyl (C-OH), epoxide (C–O–C) and single-bonded oxygen at the surface (C-O) [59,60]. The oxygen functional groups have different thermal decomposition temperatures. The thermogram of GO revealed that the decomposition occurred in different reaction steps, namely the initial step, second step, and final step. The initial step represents the rapid decline in GO weight, and this occurred before 100 °C and ended at 320 °C. The weight loss around 100 °C is due to the evolution of water (H_2_O). The water lost in this step was physically absorbed between the layers of GO. The weight loss above 100 to 320 °C is attributed to the loss of CO_x_ groups (carbon monoxide and carbon dioxide) [61]. The second step was a slow step where the GO continues to decompose, possibly due to the loss of *sp*^2^ carbon atoms in hexagonal structure that occurs between the decomposition temperatures of 320 and 645 °C. The drastic weight loss between 320 and 645 °C is caused by the loss of labile oxygen-containing functional groups such as hydroxyl and epoxy group due to their bond strength [62]. The final step exhibits a slower reduction in mass that ends at 1000 °C, signaling complete decomposition of GO to char.

All N-rGO samples were found to have different decomposition temperatures. The N-rGO synthesized at the higher doping temperatures (700 and 800 °C) showed no massive mass loss in the decomposition range of 100–300 °C, revealing the efficient removal of oxygen functional groups during the thermal process in the synthesis. All the thermograms (Figure 4) showed that N-rGO synthesized at the highest doping temperature (800 °C) were more thermally stable while samples prepared at the lowest doping temperature (600 °C) displayed the lower thermal stability. The most stable N-rGO were synthesized at doping temperature of 800 °C with decomposition temperatures of 591, 577 and 520 °C for N-rGO-1N, N-rGO-2N, and N-rGO-3N, respectively. At a doping temperature of 600 °C, N-rGO-1N, N-rGO-2N, and N-rGO-3N showed decomposition temperatures of 545, 536 and 488 °C, respectively. The nitrogen precursor 4-aminophenol produced N-rGO-1N that are more structured with fewer defects resulting in a higher thermal stability. On the other hand, the other nitrogen precursors, namely 4-nitroaniline and 4-nitro-*ο*-phenylenediamine produced, N-rGO of lower thermal stability which suggests that the samples contain a greater extent of nitrogen-doping. There is a distinct decomposition pattern of N-rGO-2N around 300 to 500 °C which is associated with the removal of stable functionalities. Similar observation was reported by Khandelwal et al. [63]. The thermal stability of N-rGO samples correlates to its lower crystallinity, which is supported with microscopic studies and Raman spectroscopy analysis (Table 2). The thermal stability of N-rGO is also associated with nitrogen bonding configuration (nitrogen functionalities) in a graphene network. Kumar et al. [64] reported that pyridinic-N configuration are mostly dominant at lower doping temperatures. However, at higher doping temperature, graphitic N is more dominant, and this results in more thermal stable N-rGO (with graphitic N). This is evidence of a temperature-dependent nitrogen configuration doping in N-rGO. Hence, it is possible to achieve selective configurative nitrogen-doping, a major breakthrough in tuning physicochemical properties of N-rGO. 

### 3.4. Surface Chemistry

#### 3.4.1. Surface Area and Porosity

The surface areas, pore volumes, and pore size distributions of N-rGO obtained at varying doping temperatures are shown in Table 3. All as-synthesized N-rGO exhibited different surface areas and pore volumes/sizes. GO had a surface area of 59.46 m^2^ g^−1^, but after thermal annealing and doping with nitrogen, the surface area and pore volume increased. Thermal annealing during doping of GO caused additional exfoliation which resulted in increased surface areas and pore volumes in N-rGO due to perforations of the sheets [65]. 

The effect of doping temperatures and nitrogen precursors on the surface areas, pore volumes, and pore sizes were investigated. It was observed that for all precursors, N-rGO synthesized at the highest doping temperature (800 °C) had a smaller surface area than N-rGO synthesized at the lowest doping temperature (600 °C). However, it has been reported that higher doping temperatures during synthesis of N-rGO create smaller nanocrystalline graphene sheets, porous structures, large surface areas and more defects [66]. In this work, the low surface area is caused by the collapse of the carbon skeleton structure during the annealing process, therefore reducing the surface area of N-rGO.

Apart from doping temperatures, the surface area was also influenced by the nitrogen content in N-rGO. The largest surface area (154.02 m^2^ g^−1^) was observed for the nitrogen precursor 4-nitro-*ο*-phenylenediamine (N-rGO-3N-600 °C) while the smaller surface area (65.05 m^2^ g^−1^) was obtained for N-rGO-1N-800 °C which was synthesized from 4-aminophenol. The high surface area in N-rGO-3N and N-rGO-2N may suggest a high percentage of pyridinic-N site in these N-rGO [67]. A good trend of BET surface area and pore sizes was observed in N-rGO samples. This illustrated that the surface area and pore volume increase with an increase in nitrogen content of N-rGO. This is because of the formation of extra pores on the surface of GO after doping, which is associated with extra exfoliation and perforation on the sheets [49,68].

**Figure 5:**
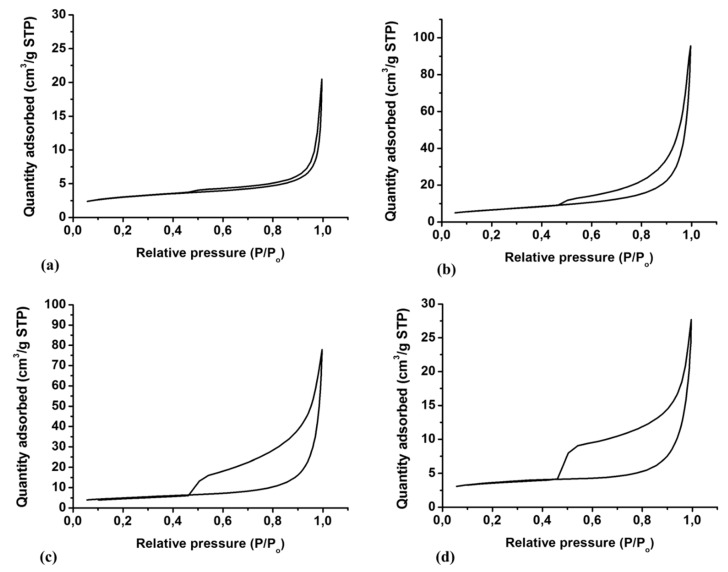
N_2_ adsorption-desorption isotherms of (**a**) GO, (**b**) N-rGO-1N-600 °C, (**c**) N-rGO-2N-600 °C and (**d**) N-rGO-3N-600 °C.

The nitrogen adsorption-desorption isotherms (Figure 5) for these materials can be classified as represent a Type IV isotherms [69]. The Type IV isotherms were accompanied by a well-defined H_3_ hysteresis loops which are associated with capillary condensation. For N-rGO-1N-600 °C, N-rGO-2N-600 °C and N-rGO-3N-600 °C, the H_3_ type hysteresis loops ranged from 0.49, 0.46 and 0.45 P/P_o_, respectively, to 1.0 P/P_o_. This demonstrates the presence of micro- and meso-porous structures within the N-rGO layers with plate-like slit-shaped pores [70].

#### 3.4.2. Functional Groups

**Figure 6:**
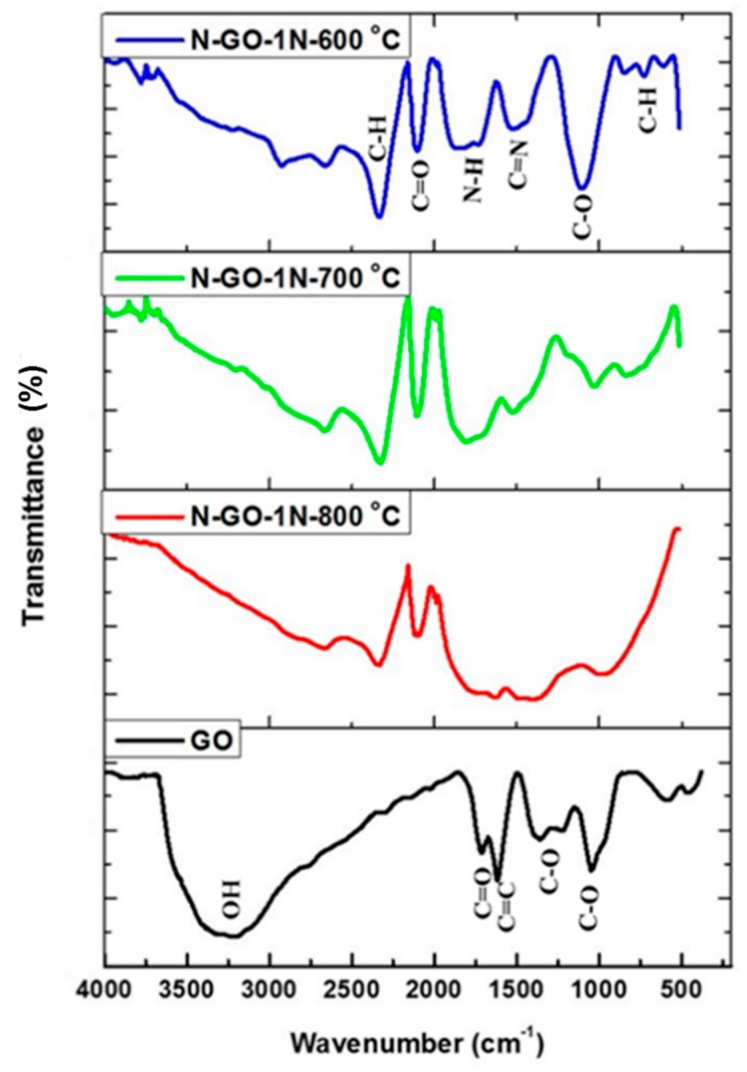
FTIR spectra of GO and N-rGO-1N at doping temperatures of 600, 700 and 800 °C.

N-rGO and GO were characterized by means of FTIR spectroscopy to investigate the effect of doping temperatures and nitrogen precursors on the functional groups present in the N-rGO. The FTIR spectral patterns of all the N-rGO samples were used to identify the presence of different functional groups, by comparison with that of GO (Figure 6).

The FTIR spectrum of GO showed different functional groups including hydroxyl (O-H), carbonyl (C=O), (C=C) and (C-O), indexed at 3157, 1733, 1614 and 1255/1155 cm^−1^, respectively, which are similar to those previously reported [71]. After nitrogen-doping of GO, peaks for C=N stretching vibrations and N-H bending vibrations occurred at 1348 and 1660 cm^−1^, respectively. Higher doping temperatures tend to reduce the peak for the O-H stretching vibration. The C-H stretching vibration peak is observed at 2489 cm^−1^ while the peak at 650 cm^−1^ (fingerprint region) is assigned to the C-H bending vibration (hybridized *sp*^2^ bonding). The FTIR spectra of N-rGO synthesized from other nitrogen precursors (4-nitroaniline and 4-nitro-*ο*-phenylenediamine) revealed the presence of similar functional groups as for N-rGO-1N synthesized from 4-aminophenol.

#### 3.4.3. Nitrogen Contents

Elemental analysis (CHNS/O) of the N-rGO samples prepared was performed to study the relationship between the nitrogen precursors and the elemental composition of N-rGO. All the synthesized N-rGO samples contained different compositions carbon, oxygen and nitrogen (Table 4). The highest doping temperature for each nitrogen precursor produced N-rGO with a lower nitrogen content, while the lowest doping temperature resulted in largest nitrogen content for all nitrogen precursors. Thus, a doping temperature of 600 °C was found to be the best temperature for the nitrogen-doping of GO. A similar trend was reported by Song et al. [56] where GO was doped by hydrothermal treatment with ammonia as the nitrogen precursor at doping temperatures of 160, 190, 220, 250 and 280 °C. Thus, the largest nitrogen content occurred at a doping temperature of 160 °C. 

Varying the nitrogen precursors was found to influence the level of doping of N-rGO. 4-nitro-*ο*-phenylenediamine which contains the most nitrogen atoms in its structure (3 N atoms per molecule), resulted in a high nitrogen content than for the other nitrogen precursors. The nitrogen content of N-rGO-3N synthesized from 4-nitro-*ο*-phenylenediamine at temperatures of 600, 700 and 800 °C was 15.431, 11.981 and 9.578%, respectively. As the nitrogen content in N-rGO increased, the oxygen content was also found to increase. The decrease in oxygen content from a doping temperature of 600 to 800 °C was attributed to deoxygenation in N-rGO. A higher oxygen content was observed in the N-rGO-3N-600 °C. This is because 4-nitro-*ο*-phenylenediamine contains more oxygen atoms in its structure than the other precursors, therefore, during the doping process, oxygen was also introduced. A correlation was observed between the crystallinity and elemental composition of N-rGO. Less crystalline N-rGO was found to contain a higher nitrogen content. This is exemplified by N-rGO-3N-600 °C with a greater density of defects (high I_D_/I_G_ ratio) and nitrogen content. An increase in nitrogen-doping also resulted in a decrease of crystallite size (Table 2). This is consistent with the findings reported by Zhang et al. [72]. 

**Figure 7:**
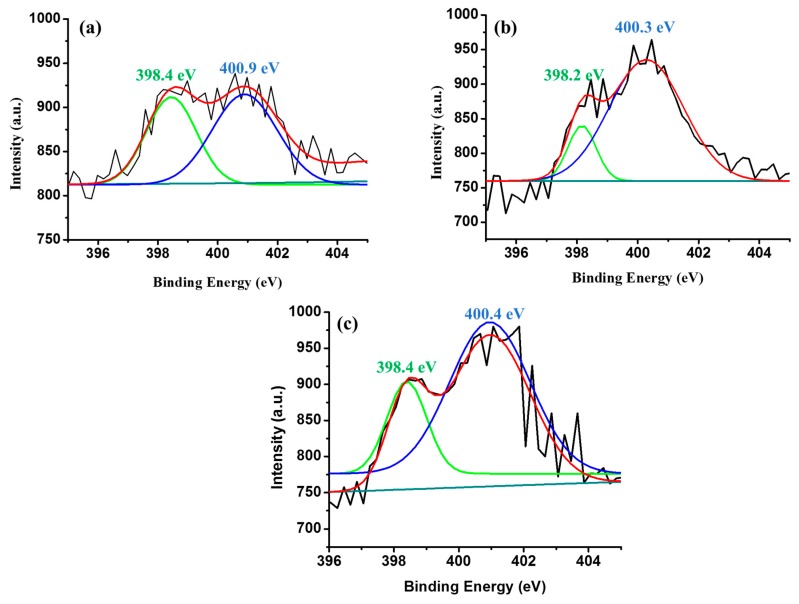
XPS high-resolution N 1s spectra of (**a**) N-rGO-1N-600 °C, (**b**) N-rGO-2N-600 °C and (**c**) N-rGO-3N-600 °C.

The nitrogen bonding configuration in N-rGO affects the electronic properties [73]. Thus, the incorporation of nitrogen in GO and the C-N bonding configurations were investigated by means of XPS (Figure 7). The trend in nitrogen content observed from the XPS analysis correlates with the results obtained from elemental analysis (Table 4). The C 1s and N 1s peaks in N-rGO appear at about 284 and 400 ± 0.1 eV, respectively. The C 1s spectra (Supplementary Materials–Table S1) of N-rGO synthesized from different nitrogen precursors show a slight shift in the binding energies of the peaks that correspond to C=C, C-N, C-O, carboxylate (O=C-O) and carbonyl (C=O) bonds. Zhang et al. [74] and Sheng et al. [75] reported that the C-O bonding configuration disappears after the doping and annealing process. However, this was not the case here since the C-O bonding peak remained, and this indicates that most oxygen groups in N-rGO were not completely removed. N-rGO-3N-600 °C showed C, O, and N peaks with percentage compositions of 81.5, 9.5 and 8.5%, respectively. N-rGO-3N-600 °C had a higher nitrogen content and lower carbon and oxygen content than N-rGO-1N-600 °C and N-rGO-2N-600 °C.

Nitrogen-doped reduced graphene oxide is reported to contain three most desired, nitrogen bonding configurations namely pyrrolic-N, pyridinic-N, and graphitic N with different components of N 1s at 399.8–401.2 eV, 398.1–99.3 eV and 401.1–402.7 eV, respectively [76,77]. However, these N 1s positions vary in comparatively wide ranges in different studies. Figure 7 shows a comparison of the different XPS spectra and the presence of various nitrogen (N 1s) species in N-rGO. The N 1s spectra of all N-rGO samples were fitted into two peaks, namely pyrrolic-N and pyridinic-N. The N 1s spectra for N-rGO-1N-600 °C and N-rGO-2N-600 °C are lower in intensity, therefore it was not possible to determine the percentage of each bonding configuration. However, the N 1s spectrum for N-rGO-3N-600 °C was fitted into two N 1s peak, namely pyrrolic-N at 400.4 ± 0.1 eV with a 46% content and pyridinic-N at 398.4 ± 0.1 eV with a 54% constant. These results suggest that N-rGO-3N-600 °C possesses a greater content of pyridinic-N than pyrrolic-N. Increase of pyridinic-N in N-rGO is related with lower thermal stability and higher surface area. This suggests that there are more defects in N-rGO-3N-600 °C. The lower pyrrolic-N content in N-rGO-3N-600 °C is due to the lower stability of pyrrolic-N which occurs in carbon materials that are doped at lower temperatures [78].

The doping temperatures control the type of nitrogen bonding configuration. For example, low doping temperatures are reported to produce N-rGO in which pyrrolic-N and pyridinic-N dominate [56]. Lu et al. [24] also noted that N-rGO synthesized at low temperatures acquired a higher degree of microstructural disorder associated with the higher nitrogen content. Moreover, it was found that the quality of the resulting N-rGO microstructure was directly dependent on the doping temperature.

### 3.5. Phase Composition

**Figure 8:**
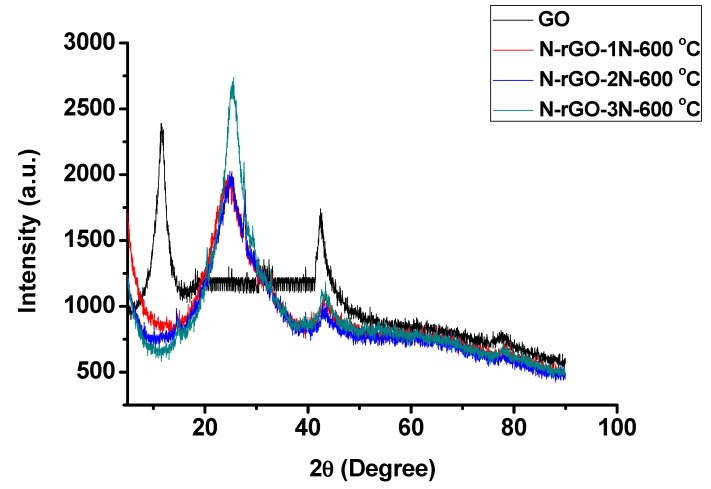
Powder X-ray diffractograms of GO, N-rGO-1N-600 °C, N-rGO-2N-600 °C and N-rGO-3N-600 °C.

The structure and phase compositions of all N-rGO were investigated by powder-XRD. The X-ray diffractograms of GO, N-rGO-1N-600 °C, N-rGO-2N-600 °C and N-rGO-3N-600 °C are presented in Figure 8. In the case of GO a diffraction peak was observed at 13.8° (2θ). However, all the samples with nitrogen are devoid of this peak at 2θ = 13.8°. This may be due to the deoxidization of oxygen-containing functional groups in the N-rGO structure. The (004) diffraction peak indicates the crystallinity of the synthesized GO and N-rGO. The diffractograms of N-rGO-1N-600 °C, N-rGO-2N-600 °C and N-rGO-3N-600 °C have a broad diffraction peak at 25.1°, 25.5° and 25.6°, respectively, indicating a high graphitic degree. The nitrogen-rich sample, N-rGO-3N-600 °C have a higher shift of the 2θ angle compared with N-rGO-1N-600 °C and N-rGO-2N-600 °C. The 2θ angle shift may be caused by strain, stress, defects, and dislocation induced in the crystal lattice during nitrogen-doping. 

Microstructural parameters (lattice dimensions, dislocation density and micro-stain) were determined from the diffraction 2θ angles and the Scherrer equation. Table 5 shows various XRD parameters for N-rGO.

The peak intensity and peak width of 2θ vary significantly depending on the doping of GO. The FWHM of 2θ of N-rGO increased with increased nitrogen content, which in turn depended on the nitrogen precursor. However, the shift in 2θ causes a decrease in the d_002_ spacing which is associated with reduction of epoxy, hydroxyl, and carboxyl functional groups on the GO framework. For instance, the broad (002) peak and decrease in crystallinity in N-rGO-3N-600 °C is due to the increased nitrogen content. Increasing the nitrogen content causes an increase in structural strain of N-rGO-3N-600 °C, thus resulting in enhanced surface defects of the graphite layer which probably led to broadening of the FWHM of the peak. The increase if FWHM observed concurred with the decrease in thermostability from TGA analysis (Figure 4). These changes also correspond to the variations in lattice distortions and d_002_ spacings. For example, the d_002_ spacing of GO (0.640 nm) was larger than those of N-rGO and this correlates with the increase in nitrogen content. N-rGO-3N-600 °C with a higher nitrogen content of 8.5% had a d_002_ spacing of 0.182 nm, while a lower nitrogen content (3.0%) in N-rGO-1N-600 °C resulted in a d_002_ spacing of 0.186 nm. The d_002_ spacings of the N-rGO samples obtained from XRD correspond with those determined from HR-TEM analysis (Table 1). Hence, it can be concluded that nitrogen-doping has an influence on the d_002_ spacing. The crystallite sizes of the synthesized N-rGO also tend to decrease with a higher nitrogen content in N-rGO, which indicates an increase in the number of defects induced. These observations of crystallite size (XRD) correlate with the calculated crystallite sizes from Raman spectroscopy (Table 2). N-rGO-3N-600 °C had a smaller crystallite size (0.75 nm) than either GO or the other N-rGO (N-rGO-1N and N-rGO-2N).

### 3.6. Optical Properties

**Figure 9:**
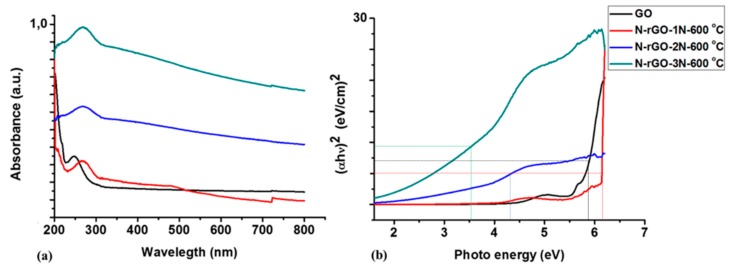
(**a**) UV-Vis absorption spectra and (**b**) Tauc plots for GO, N-rGO-1N-600 °C, N-rGO-2N-600 °C and N-rGO-3N-600 °C.

The optical properties of the N-rGO were investigated by UV-Vis spectrophotometry. From the work reported by Loryuenyong et al. [79], the maximum absorption wavelength of GO was reported to be around 230–270 nm. In Figure 9, GO exhibited a maximum absorption peak at 234 nm that is associated with π-π* and n-π* transitions of C=C and C=O bonds, respectively [80]. In contrast, N-rGO shows an absorption peak at 262–275 nm. The shift to a longer wavelength indicates the deoxygenation and restoration of the electronic π-conjugation of GO [81]. The peaks between 260–275 nm in the N-rGO spectra are attributed to *π*-π* transitions of the double bonds. The introduction of more lone electrons creates more n-π* transitions which has a tendency of shifting absorption longer wavelength (since energy is inversely proportional to the wavelength). This shows a characteristic of *sp*^2^ hybridization bands and lone pairs of nitrogen. A significant increase in absorbance was noted which shifted towards the visible light range as the nitrogen content increases. This shift also enables N-rGO to have better capability of light-harvesting compared with GO. This is because freer electrons are easier to excite than bound (*π*-electrons) and therefore *π*-π* are fewer than n-π*, hence the shift to longer wavelength, a phenomenon which is required for light-harvesting. Mohamed et al. [82] reported that heteroatom-doped graphene with an absorption frequency ranging from 300–650 nm had limited photocatalytic activity resulting in a lower light-harvesting capability. The nitrogen-rich sample (N-rGO-3N-600 °C) exhibited the slight shift in absorption peak.

In the Tauc plot for N-rGO and GO (Figure 9b), N-rGO exhibited two absorption edges which correspond to rGO. The optical band gaps obtained from the Tauc plots are: 5.9 eV for GO, 6.2 eV for N-rGO-1N-600 °C, 4.4 eV for N-rGO-2N-600 °C and 3.5 eV for N-rGO-3N-600 °C. The optical band-gap (Table 6) was also recalculated by the Planck’s quantum equation to confirm the trend displayed in the Tauc plots. N-rGO-3N-600 °C showed a slight decrease in energy band-gap (4.5 eV). While N-rGO with a lower nitrogen content; i.e., N-rGO-1N-600 °C and N-rGO-2N-600 °C had a band-gap of 4.8 and 4.6 eV, respectively. 

**Figure 10:**
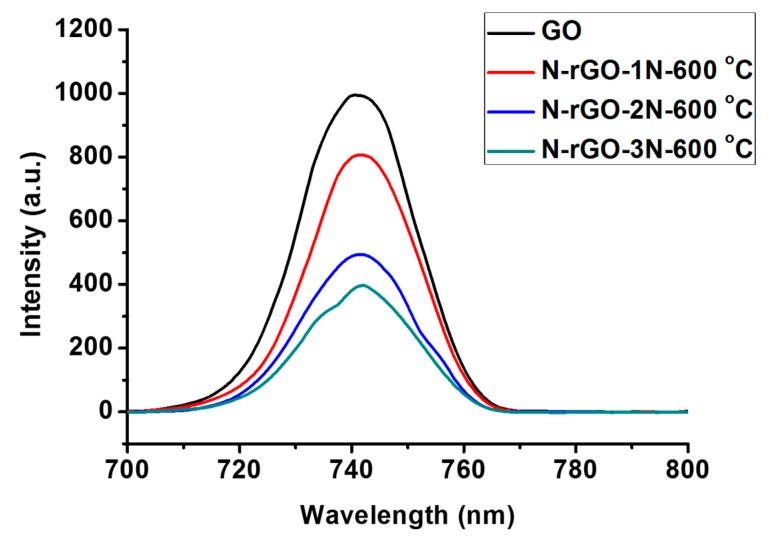
A comparison of the photoluminescence spectra of N-rGO.

The decrease in band-gap energies of N-rGO may be due to compensation of the band-gap states associated with the incorporation of dopant atoms, and this resulted in the Fermi level moving up in the direction of the conduction band edge [83]. This implies that a higher nitrogen content in N-rGO induces a lower rate of electron hole (e^−^/h^+^) recombination, than for N-rGO with a lower nitrogen content. The large band gaps for N-rGO-1N-600 °C and N-rGO-2N-600 °C indicate that lower nitrogen content may not be ideal for light-harvesting. Smaller band-gap energies can lead to enhancement of visible light trapping than larger band-gap energies. The e^-^/h^+^ recombination of N-rGO was therefore investigated by photoluminescence spectroscopy. A comparison of the e^-^/h^+^ recombination dynamics of N-rGO are presented in Figure 10. 

The intra-band-gap, which is associated with local defect functions as a trap for free carriers, affects the recombination and electron transport [84]. All N-rGO from different nitrogen precursors luminescence at e^-^/h^+^ recombination rate of 745 nm as shown in Figure 10. However, their photoluminescence peak intensities are different. The variation of nitrogen precursor showed the enhancement of photoluminescence peak intensity. Van Khai et al. [85] reported that the doping temperatures tend to cause a shift in wavelength, which is due to the presence of quaternary-N, whereas the presence of pyrrolic-N and pyridinic-N was closely related with enhancement of photoluminescence peak intensity. Therefore, the enhanced photoluminescence intensity in N-rGO corresponds to the decrease density of pyrrolic-N. However, the increased density of pyridinic-N may be correlated with the decrease of non-radiative recombination. N-rGO-1N-600 °C with a lower nitrogen content had a higher photoluminescence peak intensity while N-rGO-3N-600 °C with a higher nitrogen content was of lower intensity This further suggests that N-rGO-3N-600 °C has a lower rate of a e^-^/h^+^ recombination.

### 3.7. Electrical Conductivity Properties

**Figure 11:**
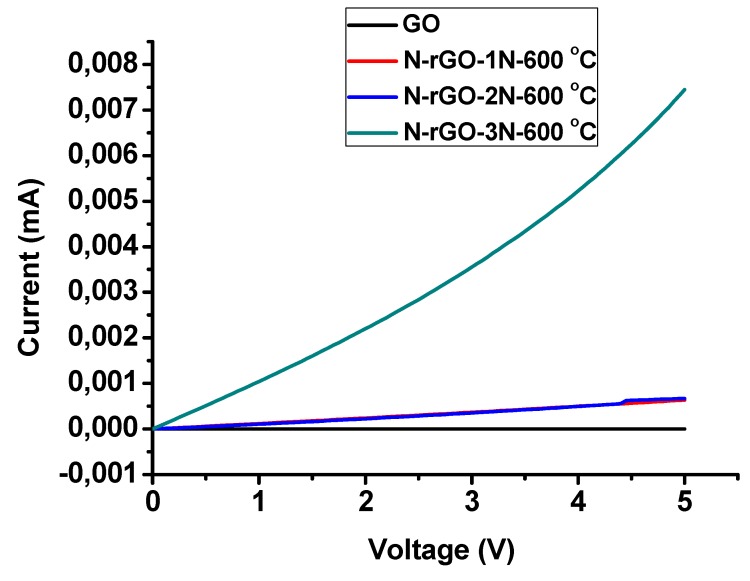
Current–voltage curve of N-rGO at doping temperature of 600 °C.

To study the effect of nitrogen content on the electrical conductivity of N-rGO, the current–voltage (I–V) characteristics (Figure 11). All the N-rGO samples exhibited a linear I-V relationship. However, the I-V slope of GO sample is almost close to zero. This is due to high oxygen content (oxygen functional group) which cause GO to behave like an insulating material [86]. Generally, the structure of GO is amorphous because of distortions from the *sp*^3^-oxygen (C–O–C, C–OH, and COOH). Additionally, because of the random dispersion, the *sp*^2^-hybridized benzene rings are isolated by *sp*^3^-hybridized rings, in this way prompting the insulating characteristics. In the case of N-rGO, the I–V slope significantly increased, demonstrating that the electrical conductivity of N-rGO was enhanced. The enhanced electrical conductivity can be attributed to reduction of oxygen functional groups and restoration of *sp*^2^ carbon network.

The electrostatic investigation on the effect of nitrogen-doping on electrical band-gap of GO was carried out by first determining resistivity (ρ), using Equation (2).

(2) $$\rho =\left(\frac{\pi }{ln2}\right)\left(\frac{V}{I}\right)t$$

where *V* is the voltage, *I* is the current, and t is the sheet thickness. The obtained *ρ* was then used in the estimation of electrical band-gap (*E_g_*) given Equation (3).

(3) $$E_{g}=2k\frac{ln\rho }{\frac{1}{T}}$$

where *k* is the Boltzmann constant (0.000086 eV/K) and *T* is the temperature in Kelvin. It was generally observed that nitrogen-doping has an effect of reducing electrical band-gap of GO structure (Table 7). However, N-rGO-1N-700 °C and N-rGO-2N-800 °C had non-linear relations an indication of two conductive regimes. This can be attributed to dominant N configuration, doping concentration, and the respective temperatures. At 700 °C doping temperature and much lower dopant concentration a pyrrolic-N configuration is dominant a higher conjugation on average is expected and therefore much lower electrical band-gap compared to the pyridinic configuration at the same temperature.

The electrical conductivities are shown in Table 8, obtained from four-point probe measurements, this is exhibiting an increase in conductivity with an increase in nitrogen content. N-rGO-3N-600 °C sample exhibited the highest electrical conductivity and excellent ultra-low electrical resistivity. So far, the mechanism of enhancement of electrical conductivity of N-rGO is not yet understood. It is believed that the interaction of N-C and interfacial structure are key variables of controlling the electrical conduction. The high electrical conductivity in N-rGO-3N-600 °C may be attributed the restoration *π*-electrons conjugated network in graphene, prompting to more formation of percolation pathways within the *sp*^2^ carbon atoms. The dominant nitrogen bonding configuration in N-rGO-3N-600 °C is pyridinic-N, which allows electron within *π*-graphene structure, lower stone-wall defects, creating high electron percolation pathways and quite conduction gap, hence, enhanced conductivity. Therefore, N-rGO-3N-600 °C that was synthesized have basal pyridinic-N than edge substitution, due to the availability of electrons within conduction space, elevating the density of state near the Dirac point. Thus, creates a specialized band around the Dirac point. The created band gives rise to a finite density of state near the Dirac point and enhance the electrical conductivity. Hence, it can be suggested that the electrical conductivity of N-rGO might be dependent on the nitrogen content which is incorporated into the structure of GO. Consequently, N-rGO-3N-600 °C serves as a promising material for various applications such as electronic and opto-electronic devices.

## 4. Conclusions

In conclusion, N-rGO has been successfully synthesized from solid nitrogen precursors (4-aminophenol, 4-nitroaniline and 4-nitro-*ο*-phenylenediamine). The incorporation of N atom into the GO lattice at various doping temperatures caused a significant effect on the physicochemical properties such as surface morphology, surface chemistry, surface area, and porosity. Microscopic studies showed a more wrinkled-like structure on N-rGO than for GO due to the presence of nitrogen atoms in the GO framework. By lowering the doping temperature, a higher nitrogen content was incorporated into the GO lattice. The nitrogen content of N-rGO varied for different nitrogen precursors. N-rGO exhibited lower thermal stability as the level of nitrogen-doping increased, due to more defects and distortions experienced in the N-rGO structure. The enhancement of surface area and high degree of disorder on N-rGO were attributed to the removal of oxygen-containing functional groups. 

N-rGO-1N-600 °C, N-rGO-2N-600 °C and N-rGO-3N-600 °C had a nitrogen content of 3.0, 3.7 and 8.5%, respectively. The nitrogen-rich precursor, 4-nitro-*ο*-phenylenediamine, lead to higher doping of N-rGO. N-rGO-3N-600 °C was found to have the highest nitrogen content of 8.5% and a high surface area of 154.02 m^2^ g^−1^ though it was less crystalline and manifested low thermal stability. The peak fitting of N 1s in all N-rGO samples produced two major components of pyridinic-N and pyrrolic-N with different nitrogen content. N-rGO showed good absorption and luminescence in the near UV region. The photoluminescence peak intensity and band-gap values were highly dependent on nitrogen content. A higher nitrogen content in N-rGO exhibited a smaller optical band-gap of 4.5 eV with lower photoluminescence peak intensity. N-rGO-3N-600 °C exhibited higher electrical conductivity of 0.133 S cm^−1^.

## Figures and Tables

**Table 1:** Comparison of the d_002_ interlayer spacing of GO and N-rGO synthesized at different temperatures and with different nitrogen precursors.

Sample	Interlayer Spacing/nm
GO	0.47 ± 0.03
N-rGO-1N-600 °C	0.39 ± 0.01
N-rGO-1N-700 °C	0.40 ± 0.02
N-rGO-1N-800 °C	0.44 ± 0.02
N-rGO-2N-600 °C	0.38 ± 0.01
N-rGO-2N-700 °C	0.39 ± 0.01
N-rGO-2N-800 °C	0.40 ± 0.02
N-rGO-3N-600 °C	0.36 ± 0.01
N-rGO-3N-700 °C	0.38 ± 0.01
N-rGO-3N-800 °C	0.39 ± 0.01

**Table 2:** Crystallinity analysis of N-rGO.

Sample	D-Band/cm^−1^	G-Band/cm^−1^	I_D_/I_G_	I_2D_/I_G_	La/nm
GO	1355 ± 1	1601 ± 1	0.82	0.0589	5.37
N-rGO-1N-600 °C	1354 ± 1	1585 ± 1	1.04	0.0199	4.20
N-rGO-1N-700 °C	1360 ± 1	1585 ± 1	0.88	0.0212	5.00
N-rGO-1N-800 °C	1363 ± 1	1575 ± 1	0.86	0.0287	5.11
N-rGO-2N-600 °C	1357 ± 1	1603 ± 1	1.08	0.0159	4.07
N-rGO-2N-700 °C	1356 ± 1	1602 ± 1	1.02	0.0207	4.31
N-rGO-2N-800 °C	1360 ± 1	1601 ± 1	0.85	0.0253	5.37
N-rGO-3N-600 °C	1355 ± 1	1606 ± 1	1.77	0.0103	2.49
N-rGO-3N-700 °C	1355 ± 1	1605 ± 1	1.40	0.0197	3.14
N-rGO-3N-800 °C	1361 ± 1	1602 ± 1	0.88	0.0212	5.00

**Table 3:** A comparison of the surface areas and porosities of N-rGO synthesized at different temperatures and with different nitrogen precursors.

Sample	Surface Area/m^2^ g^−1^	Pore Volume/cm^3^ g^−1^	Pore Size/nm
GO	59.46	0.0564	11.39
N-rGO-1N-600 °C	87.52	0.1876	15.11
N-rGO-1N-700 °C	74.98	0.1652	17.32
N-rGO-1N-800 °C	65.05	0.1083	19.84
N-rGO-2N-600 °C	110.55	0.4324	24.89
N-rGO-2N-700 °C	99.21	0.3003	26.45
N-rGO-2N-800 °C	90.34	0.2537	27.53
N-rGO-3N-600 °C	154.02	0.5029	25.96
N-rGO-3N-700 °C	130.67	0.4986	28.89
N-rGO-3N-800 °C	95.08	0.2835	34.67

**Table 4:** Elemental composition of N-rGO.

Sample	Elemental Analysis	
	Nitrogen/%	Oxygen/%
GO	-	42.985 ± 5
N-rGO-1N-600 °C	8.351 ± 5	1.496 ± 5
N-rGO-1N-700 °C	7.053 ± 5	1.255 ± 5
N-rGO-1N-800 °C	6.982 ± 5	0.464 ± 5
N-rGO-2N-600 °C	10.204 ± 5	0.789 ± 5
N-rGO-2N-700 °C	7.697 ± 5	0.613 ± 5
N-rGO-2N-800 °C	7.490 ± 5	0.239 ± 5
N-rGO-3N-600 °C	15.431 ± 5	3.475 ± 5
N-rGO-3N-700 °C	11.981 ± 5	3.029 ± 5
N-rGO-3N-800 °C	9.578 ± 5	1.082 ± 5

**Table 5:** Powder-XRD-parameters for N-rGO prepared at a doping temperature of 600 °C.

Sample	2θ/°	FWHM/β_hkl_	Interlayer Spacing/nm	Crystallite Size/nm
GO	13.8	2.12	0.640	3.93
N-rGO-1N-600 °C	25.1	7.8	0.186	1.09
N-rGO-2N-600 °C	25.5	10.8	0.183	0.79
N-rGO-3N-600 °C	25.6	11.4	0.182	0.75

*FWHW* = full width at half maximum.

**Table 6:** Energy band-gap of N-rGO from a doping temperature of 600 °C obtained from Planck’s quantum equation.

Sample	Wavelength/nm	Band-Gap Energy/eV
GO	234	5.3
N-rGO-1N-600 °C	262	4.8
N-rGO-2N-600 °C	271	4.6
N-rGO-3N-600 °C	275	4.5

**Table 7:** Effect of nitrogen-doping temperature on electrical band-gap of N-rGO.

Sample	Resistivity/Ω mm^−1^	Conductivity/mmS m^−1^	Band-Gap/eV
GO	7.89	0.127	3.240
N-rGO-1N-600 °C	4.92	0.203	2.500
N-rGO-2N-600 °C	4.63	0.216	2.403
N-rGO-3N-600 °C	5.00	0.200	2.526
N-rGO-1N-700 °C	3.29	0.304	1.866
N-rGO-2N-700 °C	5.04	0.198	2.538
N-rGO-3N-700 °C	4.93	0.203	2.502
N-rGO-1N-800 °C	4.59	0.218	2.392
N-rGO-2N-800 °C	8.03	0.125	3.269
N-rGO-3N-800 °C	4.63	0.216	2.405

**Table 8:** Electrical conductivity of the N-rGO at doping temperature of 600 °C.

Sample	Sheet Resistance/Ω sq^−1^	Bulk Resistivity/Ω cm	Electrical Conductivity/S cm^−1^
GO	9147399.8	82894.2	1.21 × 10^−5^
N-rGO-1N-600 °C	8764.1	794.6	0.00126
N-rGO-2N-600 °C	8768.8	554.9	0.00182
N-rGO-3N-600 °C	830.5	7.5	0.133

## Supplementary Materials

The following are available online at https://www.mdpi.com/1996-1944/12/20/3376/s1, Figure S1: SEM images of (**a**) GO, (**b**) N-rGO-1N-600 °C, (**c**) N-rGO-1N-700 °C, (**d**) N-rGO-1N-800 °C, (**e**) N-rGO-2N-600 °C, (**f**) N-rGO-2N-700 °C, (**g**) N-rGO-2N-800 °C, (**h**) N-rGO-3N-600 °C, (**i**) N-rGO-3N-700 °C and (**j**) N-rGO-3N-800 °C, Table S1: Atomic percentage (%) of N 1s and C 1s peak binding energy (eV).

## References

[1] Ye M., Zhang Z., Zhao Y., Qu L. (2018). Graphene platforms for smart energy generation and storage. Joule.

[2] Lee S.D., Lee H.S., Kim J.Y., Jeong J., Kahng Y.H. (2017). A systematic optimization for graphene-based supercapacitors. Mater. Res. Express.

[3] Yao Y., Ping J. (2018). Recent advances in graphene-based freestanding paper-like materials for sensing applications. Trends Anal. Chem..

[4] Bhatt K., Rani C., Vaid M., Kapoor A., Kumar P., Kumar S., Shriwastawa S., Sharma S., Singh R., Tripathi C. (2018). A comparative study of graphene and graphite-based field effect transistor on flexible substrate. Pramana-J. Phys..

[5] Priyadarsini S., Mohanty S., Mukherjee S., Basu S., Mishra M. (2018). Graphene and graphene oxide as nanomaterials for medicine and biology application. J. Nanostructure Chem..

[6] Lu G., Yu K., Wen Z., Chen J. (2013). Semiconducting graphene: Converting graphene from semimetal to semiconductor. Nanoscale.

[7] Czerniak-Reczulska M., Niedzielska A., Jędrzejczak A. (2015). Graphene as a material for solar cells applications. Adv. Mater. Sci..

[8] Liu H., Liu Y., Zhu D. (2011). Chemical doping of graphene. J. Mater. Chem..

[9] Whitby R.L.D. (2014). Chemical control of graphene architecture: Tailoring shape and properties. ACS Nano.

[10] Chen D., Tang L., Li J. (2010). Graphene-based materials in electrochemistry. Chem. Soc. Rev..

[11] Thirumal V., Pandurangan A., Jayavel R., Ilangovan R. (2016). Synthesis and characterization of boron doped graphene nanosheets for supercapacitor applications. Synth. Met..

[12] Li S., Wang Z., Jiang H., Zhang L., Ren J., Zheng M., Dong L., Sun L. (2016). Plasma-induced highly efficient synthesis of boron doped reduced graphene oxide for supercapacitors. Chem. Commun..

[13] Usachov D.Y., Fedorov A.V., Vilkov O.Y., Petukhov A.E., Rybkin A.G., Ernst A., Otrokov M.M., Chulkov E.V., Ogorodnikov I.I., Kuznetsov M.V. (2016). Large-scale sublattice asymmetry in pure and boron-doped graphene. Nano Lett..

[14] Megawati M., Chua C.K., Sofer Z., Klimova K., Pumera M. (2017). Nitrogen-doped graphene: Effect of graphite oxide precursors and nitrogen content on the electrochemical sensing properties. Phys. Chem. Chem. Phys..

[15] Xing Z., Ju Z., Zhao Y., Wan J., Zhu Y., Qiang Y., Qian Y. (2016). One-pot hydrothermal synthesis of nitrogen-doped graphene as high-performance anode materials for lithium ion batteries. Sci. Rep..

[16] Cai W., Wang C., Fang X., Yang L., Chen X. (2015). Synthesis and characterization of nitrogen-doped graphene films using C_5_NCl_5_. Appl. Phys. Lett..

[17] Wang X., Sun G., Routh P., Kim D.-H., Huang W., Chen P. (2014). Heteroatom-doped graphene materials: Syntheses, properties and applications. Chem. Soc. Rev..

[18] Rao C.N.R., Gopalakrishnan K., Govindaraj A. (2014). Synthesis, properties and applications of graphene doped with boron, nitrogen and other elements. Nano Today.

[19] Usachov D., Vilkov O., Gruneis A., Haberer D., Fedorov A., Adamchuk V., Preobrajenski A., Dudin P., Barinov A., Oehzelt M. (2011). Nitrogen-doped graphene: Efficient growth, structure, and electronic properties. Nano Lett..

[20] Kumar M.P., Kesavan T., Kalita G., Ragupathy P., Narayanan T.N., Pattanayak D.K. (2014). On the large capacitance of nitrogen doped graphene derived by a facile route. RSC Adv..

[21] Ambrosi A., Chua C.K., Latiff N.M., Loo A.H., Wong C.H.A., Eng A.Y.S., Bonanni A., Pumera M. (2016). Graphene and its electrochemistry–an update. Chem. Soc. Rev..

[22] Lin T., Huang F., Liang J., Wang Y. (2011). A facile preparation route for boron-doped graphene, and its CdTe solar cell application. Energy Environ. Sci..

[23] Poh H.L., Šimek P., Sofer Z., Tomandl I., Pumera M. (2013). Boron and nitrogen doping of graphene via thermal exfoliation of graphite oxide in a BF_3_ or NH_3_ atmosphere: Contrasting properties. J. Mater. Chem. A.

[24] Lu Y.-F., Lo S.-T., Lin J.-C., Zhang W., Lu J.-Y., Liu F.-H., Tseng C.-M., Lee Y.-H., Liang C.-T., Li L.-J. (2013). Nitrogen-doped graphene sheets grown by chemical vapor deposition: Synthesis and influence of nitrogen impurities on carrier transport. ACS Nano.

[25] Wang T., Wang L.-X., Wu D.-L., Xia W., Jia D.-Z. (2015). Interaction between nitrogen and sulfur in co-doped graphene and synergetic effect in supercapacitor. Sci. Rep..

[26] Luo Z., Lim S., Tian Z., Shang J., Lai L., MacDonald B., Fu C., Shen Z., Yu T., Lin J. (2011). Pyridinic-N doped graphene: Synthesis, electronic structure, and electrocatalytic property. J. Mater. Chem..

[27] Zhang S., Tsuzuki S., Ueno K., Dokko K., Watanabe M. (2015). Upper limit of nitrogen content in carbon materials. Angew. Chem. Int. Ed..

[28] He W., Jiang C., Wang J., Lu L. (2014). High-rate oxygen electroreduction over graphitic-N species exposed on 3D hierarchically porous nitrogen-doped carbons. Angew. Chem. Int. Ed..

[29] Park S., Hu Y., Hwang J.O., Lee E.-S., Casabianca L.B., Cai W., Potts J.R., Ha H.-W., Chen S., Oh J. (2011). Chemical structures of hydrazine-treated graphene oxide and generation of aromatic nitrogen doping. Nat. Commun..

[30] Zhao L., He R., Rim K.T., Schiros T., Kim K.S., Zhou H., Gutiérrez C., Chockalingam S., Arguello C.J., Pálová L. (2011). Visualizing individual nitrogen dopants in monolayer graphene. Science.

[31] Li N., Wang Z., Zhao K., Shi Z., Gu Z., Xu S. (2010). Large scale synthesis of N-doped multi-layered graphene sheets by simple arc-discharge method. Carbon.

[32] Zhang X., Hsu A., Wang H., Song Y., Kong J., Dresselhaus M.S., Palacios T. (2013). Impact of chlorine functionalization on high-mobility chemical vapor deposition grown graphene. ACS Nano.

[33] Li X.-J., Yu X.-X., Liu J.-Y., Fan X.-D., Zhang K., Cai H.-B., Pan N., Wang X.-P. (2012). Synthesis of nitrogen-doped graphene via thermal annealing graphene with urea. Chin. J. Chem. Phys..

[34] Wang H., Zhou Y., Wu D., Liao L., Zhao S., Peng H., Liu Z. (2013). Synthesis of boron-doped graphene monolayers using the sole solid feedstock by chemical vapor deposition. Small.

[35] Wu T., Shen H., Sun L., Cheng B., Liu B., Shen J. (2012). Nitrogen and boron doped monolayer graphene by chemical vapor deposition using polystyrene, urea and boric acid. New J. Chem..

[36] Zhou S., Liu N., Wang Z., Zhao J. (2017). Nitrogen-doped graphene on transition metal substrates as efficient bifunctional catalysts for oxygen reduction and oxygen evolution reactions. ACS Appl. Mater. Interfaces.

[37] Guo N., Xi Y., Liu S., Zhang C. (2015). Greatly enhancing catalytic activity of graphene by doping the underlying metal substrate. Sci. Rep..

[38] Du D., Li P., Ouyang J. (2015). Nitrogen-doped reduced graphene oxide prepared by simultaneous thermal reduction and nitrogen doping of graphene oxide in air and its application as an electrocatalyst. ACS Appl. Mater. Interfaces.

[39] Zabet-Khosousi A., Zhao L., Pálová L., Hybertsen M.S., Reichman D.R., Pasupathy A.N., Flynn G.W. (2014). Segregation of sublattice domains in nitrogen-doped graphene. J. Am. Chem. Soc..

[40] Wang H., Maiyalagan T., Wang X. (2012). Review on recent progress in nitrogen-doped graphene: Synthesis, characterization, and its potential applications. ACS Catal..

[41] Van Nang L., Van Duy N., Hoa N.D., Van Hieu N. (2016). Nitrogen-doped graphene synthesized from a single liquid precursor for a field effect transistor. J. Electron. Mater..

[42] Panchakarla L., Subrahmanyam K., Saha S., Govindaraj A., Krishnamurthy H., Waghmare U., Rao C. (2009). Synthesis, structure, and properties of boron-and nitrogen-doped graphene. Adv. Mater..

[43] Bao J.F., Kishi N., Soga T. (2014). Synthesis of nitrogen-doped graphene by the thermal chemical vapor deposition method from a single liquid precursor. Mater. Lett..

[44] Zhang C., Lin W., Zhao Z., Zhuang P., Zhan L., Zhou Y., Cai W. (2015). CVD synthesis of nitrogen-doped graphene using urea. Sci. China Phys. Mech..

[45] Vishwakarma R., Kalita G., Shinde S.M., Yaakob Y., Takahashi C., Tanemura M. (2016). Structure of nitrogen-doped graphene synthesized by combination of imidazole and melamine solid precursors. Mater. Lett..

[46] Wang Z., Li P., Chen Y., Liu J., Tian H., Zhou J., Zhang W., Li Y. (2014). Synthesis of nitrogen-doped graphene by chemical vapour deposition using melamine as the sole solid source of carbon and nitrogen. J. Mater. Chem. C.

[47] Hummers W.S., Offeman R.E. (1958). Preparation of graphitic oxide. J. Am. Chem. Soc..

[48] Jiang M.-H., Cai D., Tan N. (2017). Nitrogen-doped graphene sheets prepared from different graphene-based precursors as high capacity anode materials for lithium-ion batteries. Int. J. Electrochem. Sci..

[49] Yokwana K., Ray S.C., Khenfouch M., Kuvarega A.T., Mamba B.B., Mhlanga S.D., Nxumalo E.N. (2018). Facile synthesis of nitrogen doped graphene oxide from graphite flakes and powders: A comparison of their surface chemistry. J. Nanosci. Nanotechnol..

[50] Kumar N.A., Nolan H., McEvoy N., Rezvani E., Doyle R.L., Lyons M.E., Duesberg G.S. (2013). Plasma-assisted simultaneous reduction and nitrogen doping of graphene oxide nanosheets. J. Mater. Chem. A.

[51] Pham P. (2018). A library of doped-graphene images via transmission electron microscopy. J. Carbon Res..

[52] Yen M.-Y., Hsieh C.-K., Teng C.-C., Hsiao M.-C., Liu P.-I., Ma C.-C.M., Tsai M.-C., Tsai C.-H., Lin Y.-R., Chou T.-Y. (2012). Metal-free, nitrogen-doped graphene used as a novel catalyst for dye-sensitized solar cell counter electrodes. RSC Adv..

[53] Ayala P., Arenal R., Rümmeli M., Rubio A., Pichler T. (2010). The doping of carbon nanotubes with nitrogen and their potential applications. Carbon.

[54] Zafar Z., Ni Z.H., Wu X., Shi Z.X., Nan H.Y., Bai J., Sun L.T. (2013). Evolution of Raman spectra in nitrogen-doped graphene. Carbon.

[55] Matsoso B.J., Ranganathan K., Mutuma B.K., Lerotholi T., Jones G., Coville N.J. (2016). Time-dependent evolution of the nitrogen configurations in N-doped graphene films. RSC Adv..

[56] Song J.-h., Kim C.-M., Yang E., Ham M.-H., Kim I. (2017). The effect of doping temperature on the nitrogen-bonding configuration of nitrogen-doped graphene by hydrothermal treatment. RSC Adv..

[57] Capasso A., Dikonimos T., Sarto F., Tamburrano A., De Bellis G., Sarto M.S., Faggio G., Malara A., Messina G., Lisi N. (2015). Nitrogen-doped graphene films from chemical vapor deposition of pyridine: Influence of process parameters on the electrical and optical properties. Beilstein J. Nanotechnol..

[58] Mallet-Ladeira P., Puech P., Toulouse C., Cazayous M., Ratel-Ramond N., Weisbecker P., Vignoles G.L., Monthioux M. (2014). A Raman study to obtain crystallite size of carbon materials: A better alternative to the Tuinstra–Koenig law. Carbon.

[59] Pei S., Cheng H.-M. (2012). The reduction of graphene oxide. Carbon.

[60] Mowry M., Palaniuk D., Luhrs C.C., Osswald S. (2013). In situ Raman spectroscopy and thermal analysis of the formation of nitrogen-doped graphene from urea and graphite oxide. RSC Adv..

[61] Justh N., Berke B., László K., Szilágyi I.M. (2018). Thermal analysis of the improved Hummers’ synthesis of graphene oxide. J. Therm. Anal. Calorim..

[62] Chang B.Y.S., Huang N.M., An’amt M.N., Marlinda A.R., Norazriena Y., Muhamad M.R., Harrison I., Lim H.N., Chia C.H. (2012). Facile hydrothermal preparation of titanium dioxide decorated reduced graphene oxide nanocomposite. Int. J. Nanomedicine.

[63] Khandelwal M., Kumar A. (2016). One-pot environmentally friendly amino acid mediated synthesis of N-doped graphene–silver nanocomposites with an enhanced multifunctional behavior. Dalton Transactions.

[64] Kumar A., Ganguly A., Papakonstantinou P. (2012). Thermal stability study of nitrogen functionalities in a graphene network. J. Phys. Condens. Matter.

[65] Youn H.C., Bak S.M., Kim M.S., Jaye C., Fischer D.A., Lee C.W., Yang X.Q., Roh K.C., Kim K.B. (2015). High-Surface-Area Nitrogen-Doped Reduced Graphene Oxide for Electric Double-Layer Capacitors. ChemSusChem.

[66] Liu S., Peng W., Sun H., Wang S. (2014). Physical and chemical activation of reduced graphene oxide for enhanced adsorption and catalytic oxidation. Nanoscale.

[67] Yang S.-Y., Chang K.-H., Huang Y.-L., Lee Y.-F., Tien H.-W., Li S.-M., Lee Y.-H., Liu C.-H., Ma C.-C.M., Hu C.-C. (2012). A powerful approach to fabricate nitrogen-doped graphene sheets with high specific surface area. Electrochem. Commun..

[68] Fu C., Song C., Liu L., Xie X., Zhao W. (2016). Synthesis and properties of nitrogen-doped graphene as anode materials for lithium-ion batteries. Int. J. Electrochem. Sci..

[69] Sing K.S. (1985). Reporting physisorption data for gas/solid systems with special reference to the determination of surface area and porosity (Recommendations 1984). Pure Appl. Chem..

[70] Qiao X., Liao S., You C., Chen R. (2015). Phosphorus and nitrogen dual doped and simultaneously reduced graphene oxide with high surface area as efficient metal-free electrocatalyst for oxygen reduction. Catalysts.

[71] Hanifah M.F.R., Jaafar J., Aziz M., Ismail A.F., Rahman M.A., Othman M.H.D. (2015). Synthesis of graphene oxide nanosheets via modified hummers’ method and its physicochemical properties. J. Teknol..

[72] Zhang C., Fu L., Liu N., Liu M., Wang Y., Liu Z. (2011). Synthesis of nitrogen-doped graphene using embedded carbon and nitrogen sources. Adv. Mater..

[73] Zhang J., Zhao C., Liu N., Zhang H., Liu J., Fu Y.Q., Guo B., Wang Z., Lei S., Hu P. (2016). Tunable electronic properties of graphene through controlling bonding configurations of doped nitrogen atoms. Sci. Rep..

[74] Zhang W., Lin C.-T., Liu K.-K., Tite T., Su C.-Y., Chang C.-H., Lee Y.-H., Chu C.-W., Wei K.-H., Kuo J.-L. (2011). Opening an electrical band gap of bilayer graphene with molecular doping. ACS Nano.

[75] Sheng Z.-H., Shao L., Chen J.-J., Bao W.-J., Wang F.-B., Xia X.-H. (2011). Catalyst-free synthesis of nitrogen-doped graphene via thermal annealing graphite oxide with melamine and its excellent electrocatalysis. ACS Nano.

[76] Baldovino F., Quitain A., Dugos N.P., Roces S.A., Koinuma M., Yuasa M., Kida T. (2016). Synthesis and characterization of nitrogen-functionalized graphene oxide in high-temperature and high-pressure ammonia. RSC Adv..

[77] Chen F., Guo L., Zhang X., Leong Z.Y., Yang S., Yang H.Y. (2017). Nitrogen-doped graphene oxide for effectively removing boron ions from seawater. Nanoscale.

[78] Chen M., Shao L.-L., Guo Y.-X., Cao X.-Q. (2016). Nitrogen and phosphorus co-doped carbon nanosheets as efficient counter electrodes of dye-sensitized solar cells. Chem. Eng. J..

[79] Loryuenyong V., Totepvimarn K., Eimburanapravat P., Boonchompoo W., Buasri A. (2013). Preparation and characterization of reduced graphene oxide sheets via water-based exfoliation and reduction methods. Adv. Mater. Sci. Eng..

[80] Jamil A., Mustafa F., Aslam S., Arshad U., Ahmad M.A. (2017). Structural and optical properties of thermally reduced graphene oxide for energy devices. Chin. Phys. B.

[81] Vinoth R., Ganesh Babu S., Bahnemann D., Neppolian B. (2015). Nitrogen-doped reduced graphene oxide hybrid metal free catalyst for effective reduction of 4-nitrophenol. Sci. Adv. Mater..

[82] Mokhtar Mohamed M., Mousa M.A., Khairy M., Amer A.A. (2018). Nitrogen graphene: A new and exciting generation of visible light driven photocatalyst and energy storage application. ACS Omega.

[83] Marschall R., Wang L. (2014). Non-metal doping of transition metal oxides for visible-light photocatalysis. Catal. Today.

[84] Chuang C.H., Wang Y.F., Shao Y.C., Yeh Y.C., Wang D.Y., Chen C.W., Chiou J.W., Ray S.C., Pong W.F., Zhang L. (2014). The effect of thermal reduction on the photoluminescence and electronic structures of graphene oxides. Sci. Rep..

[85] Van Khai T., Na H.G., Kwak D.S., Kwon Y.J., Ham H., Shim K.B., Kim H.W. (2012). Influence of N-doping on the structural and photoluminescence properties of graphene oxide films. Carbon.

[86] Van Khai T., Na H.G., Kwak D.S., Kwon Y.J., Ham H., Shim K.B., Kim H.W. (2012). Significant enhancement of blue emission and electrical conductivity of N-doped graphene. J. Mater. Chem..

