# Supplementary material for: Effect of Doping Temperatures and Nitrogen Precursors on the Physicochemical, Optical, and Electrical Conductivity Properties of Nitrogen-Doped Reduced Graphene Oxide
Source: Materials (Basel). 2019 Oct 16;12(20):3376. doi: 10.3390/ma12203376 (PMC6829554; doi:10.3390/ma12203376)
Supplement: Supplementary file 1 [file materials-12-03376-s001.pdf]

materials

MDPI

Supplementary Materials

Effect of Doping Temperatures and Nitrogen Precursors on the Physicochemical, Optical, and Electrical Conductivity Properties of Nitrogen-Doped Reduced Graphene Oxide

Nonjabulo P.D. Ngidi, Moses A. Ollengo and Vincent O. Nyamori *

School of Chemistry and Physics, University of KwaZulu-Natal, Westville Campus, Private Bag X54001, Durban 4000, South Africa; nonjabulongidi@gmail.com (N.P.D.N.); mosesollengo@gmail.com (M.A.O.)
* Correspondence: nyamori@ukzn.ac.za; Tel.: +27-31-2608256, Fax: +27-31-2603091.

Received: 10 September 2019; Accepted: 5 October 2019; Published: date

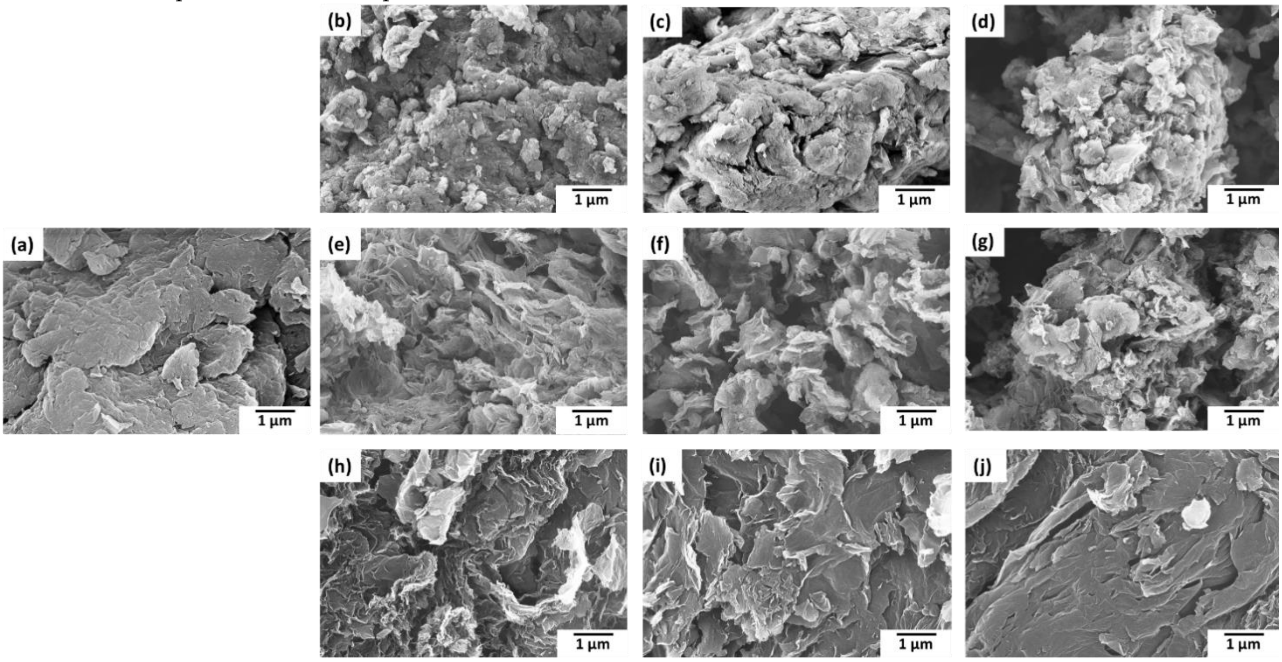

Figure S1. SEM images of (a) GO, (b) N-rGO-1N-600 °C, (c) N-rGO-1N-700 °C, (d) N-rGO-1N-800 °C, (e) N-rGO-2N-600 °C, (f) N-rGO-2N-700 °C, (g) N-rGO-2N-800 °C, (h) N-rGO-3N-600 °C, (i) N-rGO-3N-700 °C and (j) N-rGO-3N-800 °CTable S1: Atomic percentage (%) of N 1s and C 1s peak binding energy (eV).

Table S1. Atomic percentage (%) of N 1s and C 1s peak binding energy (eV).

Sample	Element	Atomic %	Peak Binding Energy (eV)	Compound (s)	Peak Area %
N-rGO-1N	C	84.5	284.8 ± 0.1 eV	C-C	76
			285.6 ± 0.1 eV	C-N	2
			286.4 ± 0.1 eV	C-O	16
			287.7 ± 0.1 eV	C=O	3
			289.0 ± 0.1 eV	O-C=O	3

materials

MDPI

	O	10.6	532.6 ± 0.1 eV	Organic O	100
	N	3.0	-	-	-
			284.9 ± 0.1 eV	C-C	77
			285.0 ± 0.1 eV	C-N	3
	C	83.7	286.5 ± 0.1 eV	C-O	14
N-rGO-2N			287.8 ± 0.1 eV	C=O	3
			289.1 ± 0.1 eV	O-C=O	3
	O	10.7		Organic O	100
	N	3.7	-	-	-
			284.7 ± 0.1 eV	C-C	76
			285.2 ± 0.1 eV	C-N	4
	C	81.5	286.3 ± 0.1 eV	C-O	14
			287.6 ± 0.1 eV	C=O	2
N-rGO-3N			288.9 ± 0.1 eV	O-C=O	4
	O	9.5	532.2 ± 0.1 eV	Organic O	100
	N	8.5	398.4 ± 0.1 eV	Organic N	54
			400.4 ± 0.1 eV	Organic N	46
